# Relationship between endoscopic gastric abnormalities and colorectal polyps: a cross-sectional study based on 33439 Chinese patients

**DOI:** 10.7150/ijms.80543

**Published:** 2023-01-22

**Authors:** Lina Feng, Kai Zhao, Ge Wang, Ruonan Dong, Mingyu Zhang, Suhong Xia, Yu Zhang, Wangdong Zhou, Dean Tian, Wei Yan, Jiazhi Liao

**Affiliations:** Department of Gastroenterology, Tongji Hospital of Tongji Medical College, Huazhong University of Science and Technology, Wuhan, Hubei, China.

**Keywords:** Colorectal polyp, *Helicobacter pylori*, Gastritis, Atrophy, Gastric polyp

## Abstract

**Background:** No study on the relationship between common abnormalities of the upper digestive tract and colorectal polyps (CPs) has been conducted.

**Methods:** 33439 patients were enrolled in this cross-sectional study, of which 7700 had available *Helicobacter pylori* (*H.pylori*) information. All participants underwent colonoscopy and esophagogastroduodenoscopy (EGD) simultaneously or within six months at Tongji Hospital, Tongji Medical College, Huazhong University of Science and Technology from January 2015 to November 2021. The study assessed whether the risk of CPs was affected by the following gastroesophageal diseases: atrophic gastritis (AG), gastric polyps, Barrett's esophagus and reflux esophagitis, bile reflux, gastric ulcer, gastric mucosal erosion, superficial gastritis, and gastric *H.pylori* infection. The crude and adjusted odds ratios (ORs) of *H.pylori* on the occurrence of CPs were computed by logistic regression. Additionally, we also evaluated whether AG had an impact on the relationship between *H.pylori* infection and CPs.

**Results:** A total of 10600 cases (31.7%) were diagnosed as CPs. Multivariate logistic analysis showed that age, male (OR, 1.80; 95% confidence interval [CI], 1.61 to 2.02), gastric polyps (OR, 1.61; 95% CI, 1.05 to 2.46 for hyperplastic polyps; OR, 1.45; 95% CI, 1.09 to 1.94 for fundic gland polyps), *H.pylori* infection (OR, 1.21; 95% CI, 1.07 to 1.37) and atrophic gastritis (OR, 1.38; 95% CI, 1.21 to 1.56) were independent risk factors for colorectal polyps. Moreover, the combined effect of *H.pylori* infection and AG was slightly greater than the sum of their individual effects on the risk of CPs, but there was no additive interaction between them.

**Conclusions:** Gastric conditions including gastric polyps, *H.pylori* infection, and AG increased the risk of CPs. However, Barrett's esophagus and reflux esophagitis, bile reflux, erosive gastritis, gastric ulcer, and superficial gastritis might not have relationship with CPs occurrence.

## Introduction

Colorectal carcinoma (CRC) is one of the most common malignancies with increasing incidence worldwide 1, 2. The 5-year survival rate of patients with early-stage CRC exceeds 90% 3, while more than 85% of Chinese patients are in advanced stage at the time of diagnosis 4. The untimely detection greatly affects the prognosis and survival of CRC patients. Colorectal polyps (CPs) are premalignant lesions associated with colorectal carcinogenesis 5. It is reported that about 85%-90% of CRC originates from polyps 6. Furthermore, one study reported that colonoscopic polypectomy reduced CRC-related long-term mortality by 53% 7. Therefore, elucidating high-risk people with CPs is very important.

In recent years, the esophagogastroduodenoscopy (EGD) examination has been more and more widely performed and has become a standard examination in clinical work. However, due to the invasiveness, inconvenience, and required preparation, the colonoscopy examination is more onerous for patients than EGD. Although some researchers have explored the relationship between *Helicobacter pylori (H.pylori)* infection, atrophic gastritis (AG), and gastric polyps and the risk of colorectal tumors or polyps, the outcomes remain controversial, and most sample sizes are relatively small. In addition, no study has investigated the association between other upper gastrointestinal diseases and the occurrence of colorectal polyps.

To solve this problem, we retrospectively analyzed the database of 33439 Chinese patients trying to find the possible association between various diseases of the upper digestive tract and CPs. We also evaluated whether AG impacted the relationship between *H.pylori* infection and CPs.

## Materials and Methods

### Study Population

**Figure 1:**
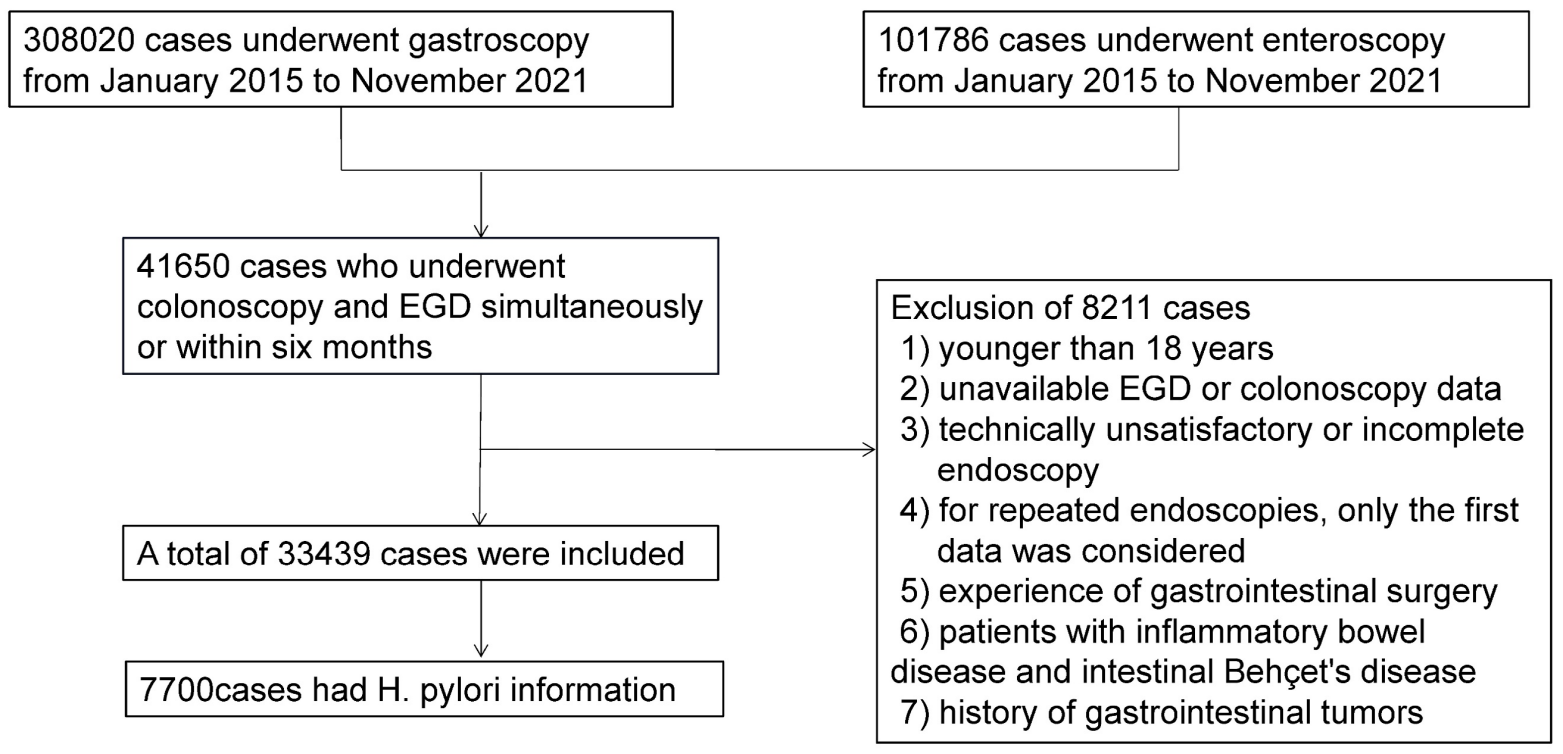
Patient flow chart.

We conducted a cross-sectional study of subjects who underwent colonoscopy and EGD at our institution from January 2015 to November 2021. During this period, 308020 cases underwent gastroscopy, and 101786 cases underwent colonoscopy. Forty-one thousand six hundred fifty subjects who had colonoscopy and EGD simultaneously or within six months were included. We excluded 8211 subjects for the following reasons: 1) younger than 18 years; 2) unavailable EGD or colonoscopy data; 3) technically unsatisfactory or incomplete endoscopy; 4) for repeated endoscopies, only the first data was considered; 5) experience of gastrointestinal surgery; 6) patients with inflammatory bowel disease and intestinal Behçet's disease; or 7) history of gastrointestinal tumors. Finally, 33439 subjects were included for analysis (Figure 1). The Institutional Ethics Board of Tongji Medical College, Huazhong University of Science and Technology (Wuhan, China) approved this study (TJ-IRB20220437). The informed consent was waived, as we solely analyzed de-identified data.

### Endoscopic impressions

At least one or two experienced gastroscopists performed gastrointestinal endoscopy examinations. Endoscopic findings were recorded as descriptive form in the endoscopy center database. EGD conditions considered relevant were the absence or presence of gastric mucosal atrophy, gastric polyps, Barrett's esophagus and reflux esophagitis, bile reflux, gastric ulcer, gastric mucosal erosion, and superficial gastritis. The diagnostic criteria of the above diseases were mainly based on endoscopic findings. In addition, 7700 subjects had available *H.pylori* information detected by urea breath test (UTB). The UTB value > 2.5% was diagnosed as *H.pylori* infection.

### Statistical Analysis

Continuous variables are summarized as means ± standard deviation (SD), while categorical variables are reported as frequency (%). Chi-squared test and Mann-Whitney U-test were used for comparison between groups. Collinearity between gastric variables was checked with variance inflation factors, and none was collinear. Univariate logistic regression was performed to identify the association between gastric findings and CPs, and the variables with *P* < 0.1 were included for further multivariate analysis. Results are calculated as odds ratio (OR) and 95% confidence interval (CI). All two-tailed P values less than 0.05 were regarded as significant. Data analyses were carried out using SPSS statistics version 23 and R 4.1.2.

Additionally, we further analyzed whether the combined effect of *H.pylori* infection and AG was greater than the sum of their individual effects on CPs. Subjects were divided into four categories according to AG and *H.pylori* status: *H.pylori* (-) & AG (-), *H.pylori* (-) & AG (+), *H.pylori* (+) & AG (-), and *H.pylori* (+) & AG (+). Using logistic regression analysis, we estimated ORs of the other three groups with *H.pylori* (-) & AG (-) group as the reference category. We evaluated the existence of additive interaction by calculating the values of synergy index (S), the attributable proportion due to interaction (AP), and the relative excess risk due to interaction (RERI) 8, as proposed by Rothman 9. The 95% CI of RERI and AP contains 0, and the 95% CI of S contains 1, indicating no additive interaction; the 95% CI of RERI and AP > 0, and the 95% CI of S > 1, indicating a positive interaction; the 95% CI of RERI and AP < 0, and the 95% CI of S < 1, indicating a negative interaction.

## Results

### General characteristics of subjects

Among 33439 enrolled cases, 10600 cases (31.7%) were diagnosed to have colorectal polyps and 22839 cases were confirmed as polyp-free controls. The mean age was 48.3±12.0 years and males comprised 58.3% of the population. The clinical characteristics of the subjects with CPs and polyp-free controls are listed in Table 1. The group of subjects with colorectal polyps was older and had a greater proportion of males, higher atrophic gastritis, higher Barrett's esophagus and reflux esophagitis, lower bile reflux, higher gastric polyps, higher gastric ulcer, higher erosive gastritis, lower superficial gastritis, and higher *H.pylori* infection as compared to the polyp-negative group.

### Analysis of risk factors associated with colorectal polyps

Table 2 contains the logistic regression results of two study populations, one is the all 33439 study subjects, and the other is 7700 cases with available *H.pylori* information. The univariate analyses of the two populations were consistent, except for bile reflux in 7700 cases (*P*=0.052). They all revealed that age, sex, gastric polyps, Barrett's esophagus and reflux esophagitis, gastric ulcer, erosive gastritis, superficial gastritis, atrophic gastritis, and *H.pylori* were significantly associated with the risk of colorectal polyps.

**Figure 2:**
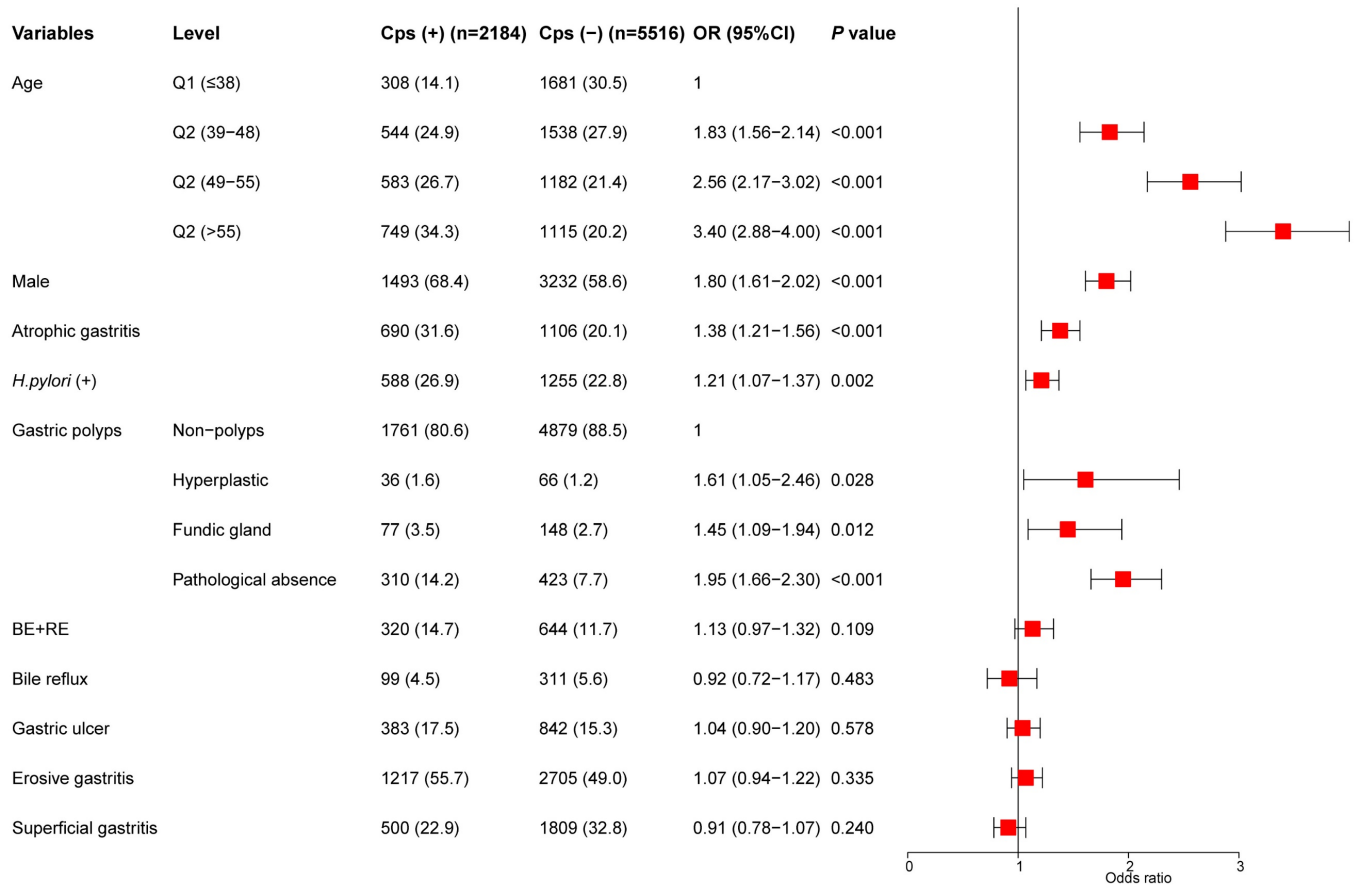
Multivariate analysis for the risk of colorectal polyps.

The indicators of univariate analysis in Table 2 (*P*<0.1) were all included in multivariate analysis. The results showed that age, sex, gastric polyps (including hyperplastic polyps and gastric fundus gland polyps), *H.pylori* infection, and AG were independent risk factors for colorectal polyps. In addition, we divided the age into four stages according to the quartile and conducted a trend test. Subjects with age in the highest quartile (> 55) had an OR of 3.40 (95% CI, 2.88-4.00) (*P* for trend < 0.001) (Figure 2).

### Separate and combined effects of *H.pylori* infection and AG on colorectal polyps

**Figure 3:**
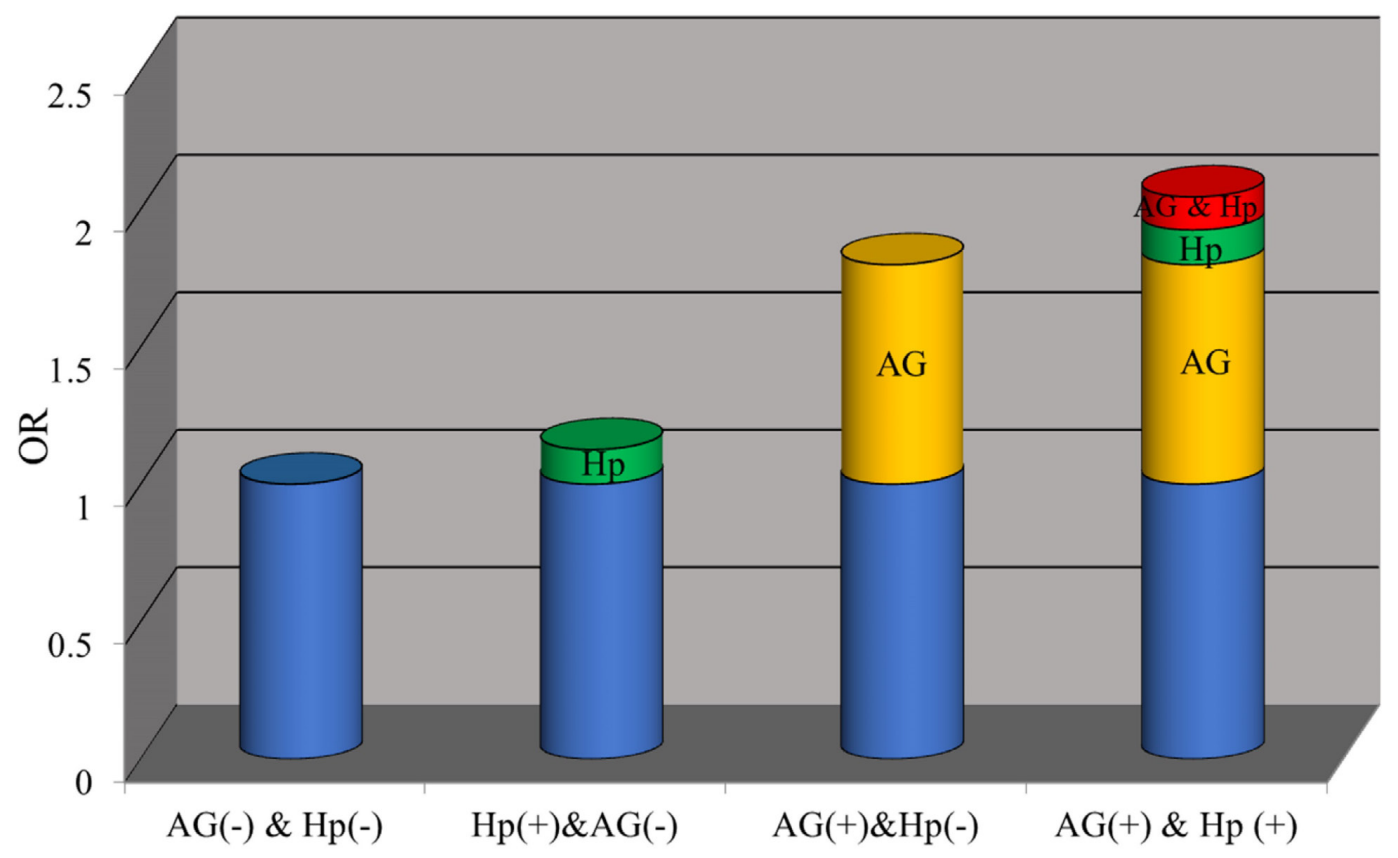
OR with contributions from different exposure categories.

As presented in Table 3, the prevalence of CPs was 24.8% in AG (-) & *H.pylori* (-), 27.3% in *H.pylori* (+) & AG (-), 37.3% in AG (+) & *H.pylori* (-) and 40.4% in AG (+) & *H.pylori* (+). Compared with subjects without *H.pylori* infection and atrophy, subjects with AG (OR: 1.80, 95%CI: 1.57-2.07) had a higher incidence of polyps, while subjects with *H.pylori*-positive (OR: 1.14, 95%CI: 0.98-1.31) did not. Moreover, subjects with *H.pylori* infection and AG increased the risk of CPs, but the relative excess risk attributable to the interaction was not statistically significant (RERI: 0.12 (-0.28-0.53), AP: 0.06 (-0.13-0.25), S: 1.13 (0.75-1.71). Figure 3 shows the separate and combined ORs of *H.pylori* infection and AG on CPs.

## Discussion

The current analysis analyzed the relationship between common gastroscopic manifestations and colorectal polyps in 33439 people. The results supported an elevated risk of CPs in individuals with gastric atrophy, gastric polyps, and *H.pylori* infection. It also showed that the risk of CPs might be further increased in subjects with concomitant AG and *H.pylori* infection.

Multiple publications have recently noticed the connection between *H.pylori* infection and CPs or CRC. However, these findings remain inconclusive. Although several research studies reported a positive association between *H.pylori* and CPs or CRC 10-13, other studies did not find any association 14-17. We speculated that the contradictory results might be partly related to different *H.pylori* detection methods, regions and races, and small sample sizes. As for the relationship between AG and CPs, several studies have shown an association between them 13, 18. Recently, Sonnenberg et al. 19 conducted a large retrospective study to assess the connection between colonic polyps and abnormal gastric histopathology, based on 302 061 patients from a national database. The data displayed that patients with gastric polyps, intestinal metaplasia, and *H.pylori* infection increased the risk of colonic polyps. Similarly, our current investigation found that *H.pylori* infection and AG were risk factors for CPs.

In addition, Lee et al. 20 found that patients with *H.pylori* infection (OR: 1.34, 95%CI: 1.04-1.72) had a higher risk of advanced CRC, especially when it coexists with AG (OR: 1.40, 95%CI: 1.03-1.91). We also found that the combined effects of *H.pylori* infection and AG were slightly greater than the sum of their individual effects on CPs. Therefore, we speculated that there might be additive interaction between *H.pylori* and AG and conducted additional analysis to verify our conjecture. Although our data showed that the additive interaction between the two on the risk of CPs was positive, it is not statistically significant. Nevertheless, we still recommend *H.pylori* eradication treatment, especially in patients with AG. Hu et al. 21 first demonstrated that *H.pylori* eradication could reduce the incidence of CPs, which needs to be confirmed in the future. Moreover, AG without *H.pylori* infection was independently associated with CPs, but *H.pylori* infection without AG was not. This result is similar to other findings 20, 22. Therefore, prolonged *H.pylori* infection may be more critical to developing CPs because most AG is usually caused by longstanding *H.pylori* infection.

Several explanations have been proposed to clarify the mechanism of positive correlation between *H.pylori* infection and CPs. First, *H.pylori* infection and AG induce hypergastrinemia 23, which might promote the development of CRC by stimulating the proliferation of colorectal mucosa 24. Several studies have shown a relationship between colorectal tumors and gastrin 25, 26, while others disagreed 27-29. Second, chronic AG induced by long-term *H.pylori* infection suppresses acid secretion. Low gastric acid may change the gastrointestinal microflora and result in bacterial overgrowth 30, 31, which may play a role in colorectal carcinogenesis. Third, as early as 1992 and 1999, it was found that viable *H.pylori* existed in the feces of infected individuals 32, 33. A few studies showed that CRC had a higher *H.pylori* detection rate compared with normal mucosa 34. Therefore, *H.pylori* might locally activate the occurrence of CRC through direct contact with the mucosa.

Moreover, we also demonstrated that gastric polyps, whether hyperplastic polyps or gastric fundus gland polyps, increased the risk of CPs, whereas Genta et al. 35 found no relation between gastric fundic gland polyps and CPs in males. However, other previous reports showed an increased risk of CPs in individuals with hyperplastic polyps and fundic gland polyps 36, 37. The existence of CPs also increased in subjects with gastric polyps in a meta-analysis (OR: 1.15, 95%CI: 1.04-1.26) 38. The relevant mechanisms are still unclear. Gastric fundus gland polyps are the main component of gastric polyps, and most of them are signs of long-term proton pump inhibitors treatment 39. Therefore, the etiology of the positive associations may relate to the reduction of gastric acid barrier. As for hyperplastic polyps, they are closely related to *H.pylori* infection and atrophic gastritis 40, 41, but whether this can explain the positive relationship is still unknown. Another suggestion is that lifestyle, environmental and genetic factors may have crucial roles in the mechanism of this correlation.

To our knowledge, male and elderly participants were at high risk of CPs and CRC. Likewise, our current research also determined an increased risk of CPs in men compared with women. Furthermore, the prevalence of CPs gradually increased with age, showing the highest OR (3.40; 95% CI, 2.88 to 4.00) in the older than 55 group.

The possible shortcomings of our study must be mentioned. Firstly, the current analysis relied almost entirely on endoscopic findings rather than histologic diagnoses, and there may be subjectivity and diagnostic variability among observers. However, our experienced endoscopists had received systematic training, and superior doctors further reviewed their uncertain examination reports. Therefore, the possibility of variability might be minimized. On the other hand, endoscopic diagnoses were also one of our advantages, increasing convenience and providing practical knowledge during daily clinical work. Secondly, we cannot obtain information about eating habits, lifestyle, disease history, medication history, and other confounding factors. However, in other studies, the associations stayed largely unaffected by these confounding factors. Thirdly, our data came from a single center in China. Thus, the generalizability of our findings needs to be confirmed by other populations. Despite these limitations, it is the first report with a relatively large sample size to investigate the relationship between gastroscopic abnormalities and CPs.

In conclusion, gastric conditions including gastric polyps, *H.pylori* infection, and AG were independent risk factors for CPs. Furthermore, *H.pylori*-infected subjects with AG might slightly enhance the risk of CPs. However, Barrett's esophagus and reflux esophagitis, bile reflux, gastric mucosal erosion, gastric ulcer, and superficial gastritis might not be related to the presence of CPs. Therefore, screening colonoscopy should be considered when patients are diagnosed as gastric polyps, AG, and *H.pylori* infection during EGD examination. For people undergoing gastroscopy, our study provides valuable evidence for physicians to recommend colonoscopy screening. In addition, further investigations are required to clarify the exact mechanism underlying the positive relationship between gastroscopic findings and CPs, and the effect of *H.pylori* eradication on susceptibility to CPs and cancer.

## Figures and Tables

**Table 1:** General characteristics of patients with and controls without colorectal polyps

	Total (n = 33439)	Colorectal polyp (+) (n = 10600)	Colorectal polyp (-) (n = 22839)	*P* value
Age, years	48.3±12.0	52.2±11.0	46.6±12.1	<0.001
Male	19499 (58.3)	7046 (66.5)	12453 (54.5)	<0.001
**Endoscopic manifestations**				
Atrophic gastritis	7129 (21.3)	2932 (27.7)	4197 (18.4)	<0.001
Gastric polyps	5859 (17.5)	2521 (23.8)	3338 (14.6)	<0.001
BE+RE	4130 (12.4)	1579 (14.9)	2551(11.2)	<0.001
Bile reflux	1758 (5.3)	474 (4.5)	1284 (5.6)	<0.001
Gastric ulcer	4122 (12.3)	1470 (13.9)	2652 (11.6)	<0.001
Erosive gastritis	15765 (47.1)	5524 (52.1)	10241(44.8)	<0.001
Superficial gastritis	11255 (33.7)	2778 (26.2)	8477 (37.1)	<0.001
*H.pylori* (+) (7700)	1843 (23.9)	588 (26.9)	1255 (22.8)	<0.001

Age is expressed as mean (SD) and all other data are expressed as number (proportion). BE, Barrett's esophagus; RE, reflux esophagitis; *H.pylori*,* Helicobacter pylori*.

**Table 2:** Univariate analysis for the risk of colorectal polyps

	Univariate analysis (n=33439)		Univariate analysis (n=7700) *	
	OR (95% CI)	*P* value	OR (95% CI)	*P* value
Age, years	1.04 (1.04-1.04)	<0.001	1.04 (1.04-1.05)	<0.001
Male	1.65 (1.58-1.74)	<0.001	1.53 (1.38-1.70)	<0.001
**Endoscopic manifestations**				
Gastric polyps	1.82 (1.72-1.93)	<0.001	1.84 (1.61-2.11)	<0.001
BE+RE	1.39 (1.30-1.49)	<0.001	1.30 (1.12-1.50)	<0.001
Bile reflux	0.79 (0.71-0.88)	<0.001	0.80 (0.63-1.00)	0.052
Gastric ulcer	1.23 (1.15-1.31)	<0.001	1.18 (1.03-1.35)	0.014
Erosive gastritis	1.34 (1.28-1.40)	<0.001	1.31 (1.18-1.45)	<0.001
Superficial gastritis	0.60 (0.57-0.63)	<0.001	0.61 (0.54-0.68)	<0.001
Atrophic gastritis	1.70 (1.61-1.79)	<0.001	1.84 (1.65-2.06)	<0.001
*H.pylori* (+)	1.25 (1.12-1.40)	<0.001	1.25 (1.12-1.40)	<0.001

BE, Barrett's esophagus; RE, reflux esophagitis; *H.pylori*, *Helicobacter pylori*. *H.pylori information available for 7700 patients.

**Table 3:** Separate and combined effects of *H.pylori* infection and AG on colorectal polyps

Exposure	N	n (%) with CPs	Crude OR (95% CI)	*P* value
AG (-) & *H.pylori* (-)	4712	1169 (24.8)	1	
*H.pylori* (+) & AG (-)	1192	325 (27.3)	1.14 (0.98-1.31)	0.082
AG (+) & *H.pylori* (-)	1145	427 (37.3)	1.80 (1.57-2.07)	<0.001
AG (+) & *H.pylori* (+)	651	263 (40.4)	2.05 (1.73-2.43)	<0.001
**Measure**	**Estimate (95% CI)**			
RERI	0.12 (-0.28-0.53)			
AP	0.06 (-0.13-0.25)			
S	1.13 (0.75-1.71)			

AG, atrophic gastritis; CPs, Colorectal polyps;* H.pylori*, *Helicobacter pylori*; RERI, the relative excess risk due to interaction; AP , the attributable proportion due to interaction; S, the synergy index.
